# Bioguided Optimization of the Nutrition-Health, Antioxidant, and Immunomodulatory Properties of *Manihot esculenta* (Cassava) Flour Enriched with Cassava Leaves

**DOI:** 10.3390/nu16173023

**Published:** 2024-09-06

**Authors:** Imane Boukhers, Romain Domingo, Axelle Septembre-Malaterre, Julien Antih, Charlotte Silvestre, Thomas Petit, Hippolyte Kodja, Patrick Poucheret

**Affiliations:** 1 Qualisud, Univ Montpellier, Avignon Université, CIRAD, Institut Agro, IRD, Université de La Réunion, 34093 Montpellier, France; imane.boukhers@gmail.com (I.B.); romain.domingo@cirad.fr (R.D.); axelle.malaterre-septembre@univ-reunion.fr (A.S.-M.); julien.antih@umontpellier.fr (J.A.); charlotte.silvestre@umontpellier.fr (C.S.); hippolyte.kodja@univ-reunion.fr (H.K.); 2 Laboratoire de Chimie et de Biotechnologie des Produits Naturels, ChemBioPro (EA2212), Université de La Réunion, 15 Avenue René Cassin, 97490 Sainte-Clotilde, France; thomas.petit@univ-reunion.fr

**Keywords:** cassava flour, cassava leaves, antioxidant effect, anti-inflammatory effect, chronic non-communicable metabolic diseases

## Abstract

*Manihot esculenta* (cassava) roots is a major food crop for its energy content. Leaves contain nutrients and demonstrate biological properties but remain undervalorized. In order to develop a bioguided optimization of cassava nutrition–health properties, we compared the phytochemistry and bioactive potential of cassava root flour extract (CF) with cassava flour extract enriched with 30% leaves powder (CFL). Cassava flour supplementation impact was explored on flour composition (starch, fiber, carotenoids, phenolic compounds), in vivo glycemic index, and bioactivity potential using macrophage cells. We assessed the impact of cassava flour supplementation on free radicals scavenging and cellular production of pro-inflammatory mediators. CFL showed higher levels of fiber, carotenoids, phenolic compounds, and lower glycemic index. Significantly higher bioactive properties (anti-inflammatory and antioxidant) were recorded, and inhibition of cytokines production has been demonstrated as a function of extract concentration. Overall, our results indicate that enrichment of cassava flour with leaves significantly enhances its nutrition–health and bioactive potential. This bioguided matrix recombination approach may be of interest to provide prophylactic and therapeutic dietary strategy to manage malnutrition and associated chronic non-communicable diseases characterized by low-grade inflammation and unbalanced redox status. It would also promote a more efficient use of available food resources.

## 1. Introduction

*Manihot esculenta* (cassava) plays an essential role in improving food security thanks to its resilience to harsh environmental conditions and year-round availability [1]. It is the main source of carbohydrates per hectare and represents the predominant source of calories for around 800 million people worldwide. Sub-Saharan Africa (SSA) is particularly dependent on cassava for its food security, with 61% of annual production coming from this region. Cassava occupies a central position in the daily diet, often replacing wheat in various forms [2,3,4,5].

However, it is essential to note that despite its importance as a source of energy (starch), its nutritional composition remains deficient in vitamins, minerals, and proteins. Therefore, a cassava-based diet may potentially contribute to nutritional deficiencies with serious consequences on human health [6,7,8,9], particularly in those parts of the world where cassava consumption is widespread [6].

Nonetheless, cassava leaves provide a different perspective. Rich in protein, minerals (Ca, Mg, P, Zn, Mn), and vitamins (B1, B2, C), they also contain interesting levels of carotenoids which, beyond their health benefits, are a precursor of vitamin A, a vitamin whose deficiency is not negligible in many countries [10,11,12,13,14,15,16,17].

Cassava leaves have the same fresh matter production yield as roots [18,19]. Several reports confirmed their nutritional potential as a complement to starch-based diets for both human and animal [12,13,20]. Nevertheless, tons of leaves are generated as waste, which, in addition to environmental pollution, results in the loss of a bioresource that could be exploited to enrich and improve a diet that may be deficient [6,13]. Cassava leaves provide significant amounts of proteins and essential micronutrients without expanding production areas, thereby respecting sustainability [13,20].

Micronutrient deficiency predominates as a form of malnutrition in low-income countries, including sub-Saharan Africa [21,22,23,24]. In this type of region, the number of people affected has risen from 181 million in 2010 to 264.2 million in 2020 [25]. This situation has serious consequences: stunted growth, weakened immune systems, infectious and inflammatory pathologies that can lead to mortality, particularly in children under 5 [26,27,28,29,30].

To a lesser degree, micronutrient deficiency is involved in other pathologies often associated with oxidative stress. These nutrients, which include vitamins and minerals, are necessary to the body’s antioxidant defenses. They maintain redox homeostasis by neutralizing excess reactive oxygen and/or nitrogen species [31].

In addition, prolonged oxidative stress generates molecular instabilities and contributes to a low-grade chronic inflammatory state. This is achieved, among other ways, through activation of nuclear factor kappa B (NF-κB). NF-κB plays a major role in the inflammatory response pathways through regulation of pro-inflammatory genes [32,33]. Chronic inflammation also disrupts tissue repair mechanisms, maintaining a continuous state of oxidative stress. Macrophages have a central role in regulating inflammation and contribute to generate pro-oxidant compounds via their metabolic activities in response to this stress [33].

This reciprocal link between oxidative stress and chronic low grade inflammation has a significant impact on various chronic diseases pathogenesis, more specifically in neurodegenerative diseases, non-communicable metabolic diseases, cancers and chronic respiratory diseases [34,35,36,37]. These conditions represent the leading cause of comorbidities, death and disability worldwide [38]. Understanding the molecular and cellular mechanisms underlying this interaction could contribute to develop therapeutic strategies aimed at disrupting this pathophysiological circle. Approaches targeting both the modulation of inflammatory responses and cellular antioxidant mechanisms could hinder the detrimental cumulative effects of these two complex processes on human health [39,40].

In addition to micronutrients, secondary metabolites—or “phytonutrients”—play an important role in maintaining and restoring redox homeostasis. This is supported by numerous in vitro and in vivo studies showing a positive correlation between their intake of these secondary metabolites and a lower risk of development of chronic non-communicable diseases [41,42,43,44].

Studies on cassava leaves have highlighted the presence of phenolic compounds and carotenoids, suggesting significant antioxidant and immunomodulatory potential [45,46,47,48].

Furthermore, studies have established a direct correlation between oxidative stress, inflammation, and hyperglycemia. This observation underlines the significant impact of diet in the prevention of chronic non-communicable diseases. Indeed, an acute rise in blood glucose levels induces a reduction in serum antioxidant concentrations and an increase in superoxide anion generated by the mitochondrial electron transport chain, thereby contributing to the generation of ROS production [49,50,51,52].

As a result, a diet associating high levels of glycemic food with low nutrient quality (particularly poor in antioxidant nutrients) is a major risk factor for the development of chronic non-communicable diseases and their comorbidities [53,54].

Cassava leaves can therefore contribute not only to the prevention of malnutrition but also act as an antioxidant, modulating inflammatory signaling pathways, thereby attenuating chronic low-grade inflammation. In addition, this vegetal matrix would contribute to the reduction in the glycemic load of a meal by providing fiber and non-carbohydrate macronutrients. Their inclusion in the diet may constitute a preventive and/or therapeutic strategy aimed at disrupting the pathophysiological mechanisms of chronic non-communicable diseases.

Therefore, the aim of the present study, through a bioguided approach, was to optimize nutrition–health properties of cassava flour with the same plant leaves. Indeed, although the composition of cassava flour is well documented in scientific literature reports, its health properties, notably its antioxidant and anti-inflammatory potential, have, to our knowledge, never been explored.

This study thus makes it possible to evaluate and compare the bioactive potential of conventional cassava flour with that of a flour enriched with a specific percentage of cassava leaves, rich in essential nutrients and bioactive secondary metabolites.

This knowledge could pave the way for integrating the intrinsic bioactivity of cassava leaves into the formulation of new food products. These products would not only be more interesting from a nutritional point of view, by filling certain types of deficiencies, but could also offer promising therapeutic potential for managing metabolic disorders, including metabolic syndrome and its associated comorbidities.

## 2. Materials and Methods

### 2.1. Plant Material

Cassava tubers low in hydrocyanic compounds were washed and peeled before being cut into pieces and dried at 45 °C for 15 h. Subsequently the dry pieces were ground, and the flour was sieved through a 100 mesh. This cassava flour constitutes the CF sample in our study and is also used for the constitution of the second study flour (CFL).

Leaves and tubers from the Republic of Guinea were collected from the same plant at 10 months of planting. Leaves were washed and dried at 45 °C before being crushed and sifted in the same way as the tubers. Enriched cassava flour (CFL) consists of 70% CF and 30% leaf powder.

### 2.2. Analysis of Phytochemicals and Glycemic Index Evaluation

#### 2.2.1. Carbohydrate Quantification

##### Starch Content

Assessment of total starch content was performed post-enzymatic degradation and D-glucose measurement. For this purpose, we used 500 mg ± 5 mg of samples that were dissolved in 30 mL of water.

Hydrolysis of starch was performed using alpha-amylase from *Bacillus licheniformis* (A3403-1MU, Sigma, Strasbourg, France). The mixture was incubated at 98 °C for 25 min in a water bath. This process was followed by an additional hydrolysis using amyloglucosidase from *Aspergillus niger* (10115, 70 U/mg, Sigma) (AMG) for 30 min at 60 °C in a water bath. D-Glucose content was assessed using a spectrophotometer (Shimadzu, Paris, France) with a reading wavelength of 510 nm. The colorimetric reaction was realized using the GOPOD reagent (glucose oxidase from *Aspergillus niger* (G6125-50kU, Sigma)/peroxidase from *Horseradish* (P8112-25kU, Sigma)). Total starch content was obtained by subtracting the quantity of simple sugars from the overall measurement.

##### Free-Form Sugars

The content of simple sugars was measured using an enzymatic approach. Briefly, 50 mg of flour was combined with 1 mL of a 5 mN sulfuric acid solution (Sigma-Aldrich, Illkirch, France). The mixture was shaken orbitally for 1 h at 20 rpm and then centrifuged at 10,000× *g*. Subsequently, 100 μL of the supernatant was mixed with 200 μL of amyloglucosidase and incubated for 30 min under ambient conditions. Following this, 2.5 mL of GOPOD reagent was added, and the mixture was incubated for an additional 20 min. The free sugars content was determined by comparing the results to a glucose calibration curve, with readings taken at 510 nm.

##### Determination of Resistant Starch

Measurement of resistant starch was carried out in accordance with AOAC method 2017.1618. Flour samples (100 mg ± 0.5 mg) were subjected to treatment with saturated concentrations of purified pancreatic alpha-amylase (PAA) and AMG. Incubation took place at 37 °C for 4 h in a linear motion shaking thermostat bath. Ethanol was added to stop the reaction, and the resistant starch pellet was isolated by centrifugation and dissolved in 2 mL of 1.7 M NaOH. Subsequent starch hydrolysis was carried out using AMG solution, with D-glucose quantified colorimetrically using GOPOD reagent at 510 nm.

##### Amylose Quantification

The ISO 2020 standard method was employed to assess amylose content [55]. In brief, and as previously described [56,57,58], 100 ± 0.5 mg of our samples (CF/CFL) were combined with 1 mL of 95% ethanol, followed by extraction with 9 mL of 2 M NaOH. The mixture was then centrifuged at 900 rpm for 10 min. A 5 mL aliquot of the supernatant was diluted with 50 mL of water, treated with 2 mL of 1 M acetic acid and 2 mL of iodine solution, and the volume was made up to 100 mL with water. The incubation was performed for 10 min at room temperature. The amylose content was measured through the absorbance reading at 720 nm.

##### Quantification of Insoluble Fiber

Insoluble fiber was quantified using the Van Soest method [56]. A sample of 1 g ± 5 mg of flour was chemically extracted using acidic and neutral detergents. For NDFs (Hemicellulose + Cellulose + Lignin), a neutral detergent solution (sodium lauryl sulfate, pH 7) was used at boiling temperature in the presence of thermostable α-amylase Termamyl^®^. For ADFs (cellulose + lignin), an acidic detergent solution (cetyl-trimethylammonium-bromide and H_2_SO_4_) (Sigma-Aldricht, Illkirch, France) was used. For ADL (lignin), samples were digested for 3 h with 72% H_2_SO_4_. Dry insoluble fibers were recovered on filters. Quantification was performed gravimetrically. The results were expressed as a percentage of the dry matter of the flour.

##### Glycemic Index (GI) Measurement

Wistar rats were housed in a controlled environment with a 12 h light–dark cycle. The room temperature was maintained at 20–22 °C via air conditioning. The animals were grouped three per cage and had unrestricted access to food (Standard A04 SAFE-Augy-France diet) and water. All procedures were conducted in compliance with French ethical standards for animal care and welfare. The criteria to exclude animals during the experiment are defined by the French & European Animal Welfare Regulation. These endpoints were used in order to extract any animal reaching one of these endpoints to comply with animal welfare. The study protocol was approved by the Languedoc-Roussillon Ethics Committee (Authorization N-APAFIS#2386-20200101409859 v3).

The GI was determined for the two types of flours. Animals were randomly assigned to a group using a random table. Each group was composed of *n* = 8 animals for all in vivo experiments with an average weight of 322.3 ± 13.2 g. Before the test, animals were fasted for an 8 h period. Two groups of animals received the two flour samples (equivalent to 2 g of carbohydrate in 2 mL/kg of rat weight). Flour samples (CF and CFL) were solubilized in water and administered to the animals using a gastric canula. A third group of animals, with an average weight of 321.5 ± 5.7, received 2 g of glucose/kg solubilized in water. The study was not subjected to potential confounders. Only the supervisor (not operators) was aware of the animal group allocation.

Glycemia evolution was monitored for 3 h. The blood glucose level was measured on one drop of tail blood every 15 min using a Contour Bayer Glucometer (Bayer Pharmaceuticals, Paris, France). Glycemic index (GI) was calculated by the ratio of the incremental area under the curve of the test food to the incremental area under the curve of the reference food and expressed as a percentage.

### 2.3. Analysis and Measurement of Bioactive Phytochemicals

#### 2.3.1. Total Phenolic Content Assay (TPC)

Flour dry extracts were used to assess TPC. Potential bioactive compounds of interest were extracted by dispersing 50 g of flour with 200 mL of an 80:20 ethanol/water solution (*v*/*v*). The mixture was subjected to sonication for 30 min at 35 °C. (ultrasonic bath Type VWR USC 300 TH). The solution was then filtered to produce Extract 1. On the retentate, a similar additional extraction was performed generating the Extract 2. A final extraction was carried out with an 80% methanol solution, yielding Extract 3. Supernatants of the 3 extractions were combined. The solution was evaporated to yield a dry extract, which was subsequently used to evaluate biological activities, including antioxidant and anti-inflammatory properties.

The TPC assay was conducted using the Folin–Ciocalteu reagent, following the previously described method [57]. A 4 mg/mL sample of the final dried extract was added with dimethyl sulfoxide (DMSO) (Sigma-Aldricht, Illkirch, France) and diluted in water to reach a 1 mg/mL concentration. A calibration curve was constructed using gallic acid concentrations ranging from 1.56 to 75 μg/mL. The TPC assay was performed on a 96-well plate where 50 μL of either the extract or gallic acid standard was added in triplicate to each well. Following this, 50 μL of distilled water was added, followed by 10% Folin–Ciocalteu reagent and 50 μL of a 1 M sodium carbonate solution. Absorbance was measured after 60 min (in the dark) at 650 nm using a microplate reader (Thermo Fisher, Paris, France). Results were reported as milligrams of gallic acid equivalents (GAEs) per gram of extract.

#### 2.3.2. Carotenoid Identification and Quantification

The extraction of carotenoids from our flour samples was carried out as previously described [58]. In short, a three-stage extraction process using an ethanol/hexane solution (4:3, *v*/*v*) was applied to isolate the carotenoids. Anhydrous sodium sulfate was used to dry the hexane phase before filtration followed by vacuum evaporation of the solution. A mixture of 80:20 (*v*/*v*) methyl tert-butyl ether (MTBE) and methanol was then added to the carotenoid extract. The resulting solution was then analyzed by HPLC using a diode array detector and a C30 column (250 × 4.6 mm i.d., 5 µm; YMC, Tokyo, Japan) for the separation of carotenoids. The column temperature was set at 25 °C. The injection volume was 20 µL. The mobile phase gradient was composed of H_2_O as eluent A, methanol as eluent B, and MTBE (methyl-tert-butyl-ether) as eluent C with a gradient. Flow rate was set at 1 mL/min. The absorbance was read at wavelength 450 nm. The Agilent Chemstation software (B.04.0x, Agilent, Paris, France) was used to treat chromatographic data and UV–visible spectra.

#### 2.3.3. Quantification of Mineral Matter (Ash)

The content of mineral materials was quantified by ashing the sample at 500 °C using an ash furnace (Thermo Scientific™ Thermolyne™ 6000 series 408, Waltham, MA, USA). The sample used was obtained following fractionation and quantification of fibers. Gravimetrical quantification was performed on the material obtained after mineralization.

### 2.4. Analysis of Bioactive Properties

#### 2.4.1. Evaluation of Antioxidant Activity

Antioxidant activity was evaluated in vitro using three assays: Nitric Oxide (NO) scavenging ability, DPPH (2,2-diphenyl-1-picrylhydrazyl) radical scavenging capacity, and antioxidant capacity measured by the ORAC test.

##### Measurement of Nitric Oxide (NO) Scavenging Potential

To assess the nitric oxide scavenging ability of CF and CFL, both samples were tested at concentrations of 100 and 50 µg/mL. The sample solutions were incubated with an equal volume of 5 mM of sodium nitroprusside solution in Hanks’ Balanced Salt Solution (HBSS) for 2 h at room temperature under light exposure. The Griess method was used to measure NO levels under its nitrite form. A standard curve of NaNO_2_ (1.56 to 100 µM) was realized to obtain numerical values. The absorbance was measured at 550 nm, and the results were reported as the percentage of NO scavenging.

##### DPPH Radical Neutralization Test

DPPH assay was performed according to the method previously described [57]. DMSO was used to solubilize CF and CFL extracts (4 mg/mL). The Trolox standard curve was prepared at various concentrations (75, 50, 25, and 12.5 µM). Ethanol was used as the blank, and chlorogenic acid (0.01 mg/mL) was used as the positive control.

The assay was performed on a 96-well plate. Extracts or positive control were deposited in each well (100 µL). For each extract, the assay was run in triplicate. A volume of 25 µL of extemporaneously prepared DPPH solution (0.4 mg/mL) was mixed with 75 µL of absolute ethanol into each well followed by a 30 min incubation at room temperature and protected from light. Absorbance reading was realized at 550 nm with a microplate reader (MDS Inc., Toronto, ON, Canada). Results were expressed as Trolox equivalents (TE µmoles per gram of dry extract) and as a percentage of inhibition (% inhibition).

##### ORAC (Oxygen Radical Absorbance Capacity) Assay

The ORAC assays were executed according to the method described in prior research [57]. Samples were solubilized in DMSO at a concentration of 1 mg/mL before being diluted to 25 µg/mL using phosphate buffer at pH 7.4 In the 96-well microplate (opaque polypropylene), 20 µL of Trolox solutions at concentrations of 0.6, 25, 12.5, 25, 50, and 75 µM, along with chlorogenic acid (0.01 mg/mL) or extracts at 25 µg/mL, were added. Subsequently, 100 µL of fluorescein solution (0.1 µM in phosphate buffer) and 100 µL of phosphate buffer were added. The plate was incubated at 37 °C for 10 min with continuous shaking. The reaction was initiated by adding 50 µL of 2,2′-Azobis(2-amidinopropane) dihydrochloride (AAPH). Fluorescence measurements were taken at an excitation wavelength of 485 nm and an emission wavelength of 535 nm over 70 min using a Tristar LB 941 microplate reader (Berthold Technologies GmbH & Co. KG, Bad Wildbad, Germany). ORAC values were derived from the regression equation relating Trolox concentration to the area under the curve of fluorescein degradation and expressed as micromoles of Trolox equivalents per gram of dry extract.

#### 2.4.2. Evaluation of Immunomodulatory Anti-Inflammatory Bioactivity

##### Macrophage Culture

The J774.A1 macrophage cell line (ATCC, TIB67) was sourced from LGC Standards (Molsheim, France). Cells were maintained in RPMI 1640 Gluta-MAX^®^ medium, enriched with 100 µg/mL of streptomycin, 100 units/mL of penicillin, and 10% inactivated fetal calf serum. Cultures were kept at 37 °C in a 5% CO_2_ incubator with 95% humidity.

##### Determination of Cell Viability via MTS/PMS Assay

Cells were plated at a density of 6 × 10^5^ cells per well in a 96-well culture plate with complete RPMI medium. Following a 20 h incubation at 37 °C with varying concentrations of extracts (25, 50, 75, and 100 μg/mL), 20 μL of MTS solution combined with PMS in HBSS was added to each well. After a further 4 h incubation, absorbance was measured at 490 nm using a microplate reader (Thermo Fisher, Paris, France), as previously described [58].

##### Evaluation of the Production of NO, IL-6, TNF-α, MCP-1, and PGE-2

J774.A1 macrophages were cultured in a 24-well plate with complete RPMI medium. Cells were pre-treated with CF and CFL extracts at concentrations of 25, 50, 75, and 100 µg/mL for 4 h. Following pre-treatment, cells were stimulated with 100 ng/mL of LPS (Lipopolysaccharide, *E. coli*, 555B5) and 10 ng/mL murine interferon-gamma (IFN). The cultures were then incubated for 16–18 h at 37 °C. Subsequently, supernatants were collected for analysis of nitrite, IL-6, TNF-alpha, MCP-1, and PGE-2.

##### Determination Nitric Oxide Production

Nitrite levels, indicative of nitric oxide production, were assessed in the culture media. Griess reagent (100 µL) was mixed with 100 µL of supernatant in a 96-well plate, followed by a 10 min incubation at room temperature. The nitrite concentration was determined by measuring absorbance at 550 nm, using a NaNO_2_ standard curve (1.56 to 100 µM). Results were expressed as percentage inhibition.

##### IL-6 (Interleukin 6) Assay

IL-6 production was measured using an IL-6 ELISA kit (Mouse IL6 ELISA; Thermo Fisher Scientific, Illkirch-Graffenstaden, France). After a pre-treatment period of 18 h with CF and CFL extracts at concentrations of 25, 50, 75, and 100 µg/mL, cells were exposed to 100 ng/mL of LPS (*E. coli*, 555B5) and 10 ng/mL of mouse IFN-gamma for 4 h. IL-6 levels in the supernatants were determined following the ELISA kit instructions. Results for IL-6 and other pro-inflammatory cytokines were reported as percentage inhibition values.

##### TNF-Alpha (Tumor Necrosis Factor Alpha) Assay

TNF-alpha levels were quantified as previously described [58]. Following a 3 h pre-treatment with CF and CFL extracts at concentrations of 25, 50, 75, and 100 µg/mL, cells were stimulated with 100 ng/mL LPS (*E. coli*, 555B5) and 10 ng/mL mouse IFN-gamma for 4 h. TNF-alpha levels in the supernatants were measured using a sandwich ELISA assay.

##### MCP-1 (Monocyte Chemoattractant Protein-1) Assay

MCP-1 production was assessed using an ELISA kit (Mouse CCL2 (MCP-1) ELISA kit; Thermo Fisher Scientific, France). Supernatants were collected 18 h post-stimulation with LPS/IFN-gamma, and diluted 1:10 in ELISA buffer to fall within the calibration range. MCP-1 levels were determined according to the kit’s instructions.

##### PGE-2 (Prostaglandin) Assay

Assay of PGE-2 in culture supernatants was performed, as previously described [58], using a competitive ELISA approach with Cayman PGE-2 ELISA KIT Monoclonal (Cayman Chemical, Ann Harbor, MI, USA), after CF and CFL pretreatment and LPS/IFN-gamma stimulation of macrophages.

### 2.5. Statistical Analysis

Statistical analysis was conducted using XLSTAT software (version 2019.4.1, Addinsoft, Paris, France). Data were presented as mean ± standard deviation from three replicates per experiment and analyzed by one-way ANOVA, with Tukey’s test for post hoc comparisons to identify significant differences (*p* < 0.05).

## 3. Results

### 3.1. Analysis of Phytochemicals and Glycemic Index Evaluation

The results of physico-chemical analyses of cassava flour (CF) and cassava flour blended with 30% leaves (CFL) are presented in Table 1, with contents expressed as a percentage of flour dry matter or starch. The CF yield was 32.19%, while the moisture content did not exceed 8.32%, below the recommended moisture content of 13%.

Cassava leaves were grown on the same plants as the roots used to produce the flour. The dried leaf yield reached 24.7%, with a moisture content of 7.12%.

As for the carbohydrate content of CF, it revealed a total content of 84.1%, mainly made up of starch, which accounted for over 98% of this amount, or 82.9 g/100 g. Amylose was measured at 20.4% of total starch, while the amount of free sugars was very low, at just 1.19 g/100 g flour.

When comparing CF to CFL results, it should be noted that the total amount of carbohydrates is statistically different, being precisely 29.57% lower than in CF. It is also remarkable that the amounts of free sugars in both flours remain negligible, with similar levels of amylose in relation to the percentage of starch.

However, as far as resistant starch is concerned, measured after a 4 h enzymatic digestion, it represents 13.14 g/100 g and 9.09 g/100 g of flour dry matter for CF and CFL, respectively, i.e., about 15% of the total starch content for both flours.

The results of insoluble fiber quantification, presented in Table 1, show a significant increase in the total quantity in CFL as expected. In fact, the amount of insoluble fiber in the enriched flour was four times higher. Specifically, the hemicellulose, cellulose, and lignin contents are 5.01%, 3.40%, and 6.79%, respectively, while the values in conventional flour do not exceed 1.92%, 1.60%, and 0.13%.

Overall, these observations demonstrate the potential of CFL for an increase in fiber intake thanks to the addition of cassava leaves, and a reduction in the proportion of carbohydrates compared to flour made exclusively from tubers (CF).

The glycemic index of the flours was measured in vivo, using a glucose solution as a reference. The results, presented in Table 1, reveal a significant difference between the two flours. Indeed, CF glycemic index is 91, while CFL index is 72, a reduction of 20.9%.

### 3.2. Analysis and Measurement of Bioactive Phytochemicals

Result analysis of potentially bioactive compounds present in the two flours studied: conventional cassava flour (CF) and conventional cassava flour enriched with 30% cassava leaf powder (CFL), are presented in Table 2. The total amount of polyphenols (TPCs) is given in mg GAE/g dry matter (DM), the results for total mineral content are expressed in g/100 g dry matter (% DM), while the quantities of carotenoids (lutein and beta-carotene) are expressed in mg/100 g (% DM).

The quantity of phenolic compounds is determined by the Folin–Ciocalteu reagent in flour extracts. The results show a significant difference between CF and CFL extracts, with a 5.7-fold higher content in the enriched flour (CFL).

Mineral content was determined using the Van Soest method in both flours. The results follow the same trend as those for phenolic compounds, with a content of 2.4 g/100 g and 4.01 g/100 g for CF and CFL, respectively.

As far as carotenoids are concerned, extraction followed by HPLC assay revealed their total absence in cassava flour (CF). However, results indicate the presence of lutein and beta-carotene at significant concentrations reaching 7.82 mg/100 g and 10.53 mg/100 g in CFL flour, respectively.

These observations show a significant increase in the content of micronutrients with nutritional and possibly bioactive properties in flour enriched with 30% cassava leaves (CFL).

### 3.3. Antioxidant Bioactivity

The antioxidant properties of CF and CFL flour extracts were evaluated using nitric oxide (NO) scavenging, DPPH, and ORAC assays, with the findings detailed in Table 3. NO scavenging activity is indicated as a percentage of inhibition across varying extract concentrations (100 and 50 µg/mL). The ORAC results are reported as micromoles of Trolox equivalents per gram of extract dry weight (µmol TE/g EDW), while DPPH data are presented both as µmol TE/g EDW and as a percentage of free radical inhibition at an extract concentration of 1 mg/mL.

The concentration-dependent nitric oxide (NO) scavenging activities of CF and CFL extracts are reported in Table 3. NO is an essential signaling molecule in the human body, but it contributes to undesirable inflammatory and oxidative processes when reaching excessive levels. These results reveal a significant increase in free radical scavenging capacity as a function of concentration for both extracts. However, notable differences are observed between CF and CFL at the same concentrations. At a concentration of 100 µg/mL, the inhibition rate was 11.2% for CF and 58.8% for CFL extract. Similarly, at a concentration of 50 µg/mL, CF showed an inhibition rate of 7.83%, while CFL showed an inhibition rate of 30.7%. This indicates a greater activity of the enriched flour (CFL) against free radicals provided by cassava leaves when compared to CF.

DPPH test results, expressed in Table 3 as a percentage of free radical inhibition and in Trolox equivalent, reveal a significantly higher free radical scavenging activity of CFL extract. At a concentration of 1 mg/mL, the CFL extract shows an inhibition rate of 60.5%, equivalent to 93.5 µmol TE/g dry extract (EDW), while the conventional cassava flour CF extract shows an inhibition of only 12% at the same concentration, corresponding to 15.6 µmol TE/g dry extract. These results confirm previous observations obtained with the NO radical scavenging assay.

ORAC test results presented in Table 3 show a similar trend to NO and DPPH tests. Indeed, CFL extract shows a significantly higher antioxidant activity than CF, reaching 3.5 times the ORAC value of the latter.

Altogether, it is therefore relevant to note that, based on all antioxidant assays results, the incorporation of 30% cassava leaves into conventional cassava flour provides a significant increase in antioxidant properties.

### 3.4. Immunomodulatory Anti-Inflammatory Bioactivity

**Figure 1:**
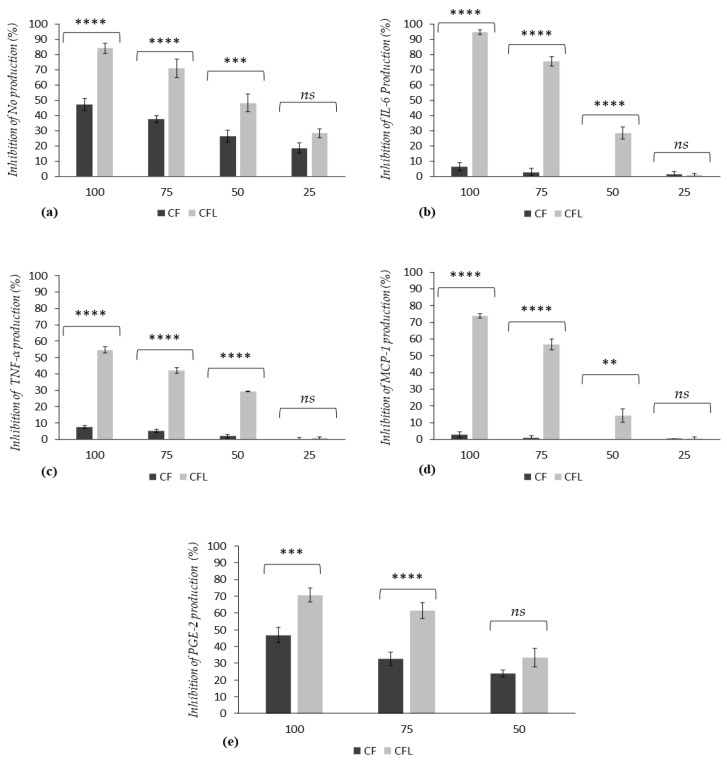
Comparison of the effect of CF and CFL extracts at different concentrations (100, 75, 50, and 25 µg/mL) on the production of NO (**a**) and proinflammatory cytokines IL-6 (**b**), TNF-alpha (**c**), MPC-1 (**d**), and PGE-2 (**e**) by J 774 inflammatory macrophages. The values measured by the Griess/Elisa reagent are expressed as the mean of the percentage inhibition of production plus or minus the standard deviation (*n* = 3) (significance: ns: not significant: *p* ≤ 0.05, **: *p* ≤ 0.01, ***: *p* ≤ 0.001, ****: *p* ≤ 0.0001).

The inhibitory potential of CF and CFL extracts on the production of inflammatory mediators (TNF-alpha, NO, MCP-1, IL-6 and PGE-2) by stimulated macrophages was investigated across various concentrations (100, 75, 50, and 25 µg/mL) to assess anti-inflammatory activity. Macrophage viability assays confirmed the non-toxic nature of the extracts at these concentrations, ruling out cytotoxicity or reduced cell numbers as reasons for changes in inflammatory marker levels. The results are displayed in Figure 1, expressed as a percentage of inhibition compared to stimulated but untreated control cells (C-LPS/IFN).

In each experiment, unstimulated and unpretreated controlled cells (C) validate the integrity of cells used in the study.

The results of nitric oxide (NO) cells production are shown in Figure 1a. This graph illustrates the levels of inhibition of macrophage NO production in response to increasing concentrations of CF and CFL extracts (25 to 100 µg/mL).

The results indicate significant reductions of NO radical production for both extracts. Inhibition was more pronounced in cells pretreated with CFL extract than in those pretreated with CF extract. Specifically, inhibition values are statistically different at concentrations of 100 µg/mL, 75 µg/mL, and 50 µg/mL, with CF showing inhibition rates of 47.2%, 37.5%, and 26.3%, respectively, while CFL extract shows inhibition rates of 84.1%, 70.9%, and 48.2%. No statistical difference was observed at a concentration of 25 µg/mL, although the absolute value of inhibition was still higher with CFL extract. These results thus indicate a concentration-dependent inhibitory bioactivity on pro-inflammatory radical nitric oxide production by macrophage cells stimulated and pretreated with CF and CFL meal extracts.

Results regarding interleukin-6 (IL-6) cells production are shown in Figure 1b. The graph illustrates the levels of inhibition of IL-6 production in the presence of CF or CFL at different concentrations (100, 75, 50, and 25 µg/mL). Statistically significant differences between the two samples were observed at concentrations of 100, 75, and 50 µg/mL, while inhibition at 25 µg/mL was almost negligible for both extracts. However, it is essential to note that an important difference is observed between the two extracts. Indeed, the results reveal an inhibition of 94.6% and 75.6% at 100 µg/mL and 75 µg/mL, respectively, for the CFL extract, while these same concentrations show an inhibition of only 6.4% and 2.88% for CF. Consequently, it was shown that increasing concentrations of extracts from both flours are associated with an increasing level of inhibition of IL-6 production, which is significantly higher for the flour enriched with cassava leaves. This observation indicates a more pronounced concentration-dependent inhibitory bioactivity of enriched flour compared to conventional cassava flour on the production of interleukin-6; a pro-inflammatory cytokine involved in the acute phase of inflammatory processes.

Results for tumor necrosis alpha (TNF-α) cells production are shown in Figure 1c. The graph represents the levels of inhibition of TNF-α production as a function of increasing concentrations of CF and CFL extracts (from 100 to 25 µg/mL). These results show highly significant differences between the two extracts at concentrations of 100, 75, and 50 µg/mL. Indeed, at the higher concentration, the inhibition value exceeds 54% for CFL, while it remains below 8% for CF extract. As for IL-6, inhibition at 25 g/mL is close to 0 for both extracts. Therefore, the addition of 30% cassava leaves induced a significant increase in the concentration-dependent inhibitory activity of cassava flour on TNF-α production, a pro-inflammatory cytokine playing a crucial role in the acute phase and systemic inflammatory processes.

Figure 1d shows the results of inhibition of monocyte chemotactic protein-1 (MCP-1) production by LPS/IFN-stimulated macrophages, exposed to different concentrations of CF and CFL (100, 75, 50, and 25 µg/mL). The results concur with those obtained for IL-6 and TNF-α cytokines. CF extract shows very low inhibition when compared to CFL at all concentrations. These results also indicate a concentration-dependent inhibition of MCP-1 cell production since increasing inhibition of MCP-1 production with increasing CFL extract concentration, from 25 to 100 µg/mL.

Figure 1e illustrates the results of inhibiting prostaglandin E2 cell production with decreasing concentrations of extracts (100, 75, and 50 µg/mL). At concentrations of 100 and 75 µg/mL, the inhibition percentages of CFL and CF extracts showed highly significant statistical differences, with the CFL extract achieving 70.7% inhibition at 100 µg/mL and 61.6% inhibition at 75 µg/mL, compared to 46.7% and 32.6% for the CF extract, respectively. At a concentration of 50 µg/mL, no significant difference was detected between the two extracts, although the absolute inhibition value was higher for the CFL extract.

It should be noted that PGE-2 is a major prostanoid playing a crucial role in regulating the homeostasis of inflammatory processes. These observations indicate a concentration-dependent inhibition of PGE-2 production for both extracts, with a more significant inhibition capacity demonstrated for cassava flour (CFL).

## 4. Discussion

The present study was conducted to explore the nutritional and bioactive potential of cassava flour, as well as to assess the impact of leaf addition on its properties.

Cassava is a fundamental drought-resistant food crop, grown in tropical and subtropical regions, widely consumed in areas where undernutrition is prevalent. It is an essential source of energy for developing countries. Cassava roots are rich in carbohydrates, while the leaves, although undervalued, contain proteins, vitamins, and minerals. Given that cassava has limited post-harvest shelf life (about three days), processing it into flour is one of the best alternatives to guarantee a stable food supply. Leaves could be dried simultaneously, enabling tuber flour to be enriched with dried leaf powder without requiring significant additional resources.

The aim of the present research was to determine to what extent enriching conventional cassava flour with up to 30% leaves could improve its nutritional value and bioactive potential. Our endeavor was intended to pave the way for innovative products conception, whether food or nutraceuticals, aiming at improving nutrition and preventing nutritional deficiencies, while contributing to the management of metabolic conditions associated with oxidative stress and low-grade inflammation.

Although previous studies have examined the sensory profile of products made from leaf-enriched cassava flour [59,60], characterization of the bioactivity of cassava roots and flour, particularly with regard to their antioxidant and immunomodulatory properties, remains limited to our knowledge. As for the leaves, recent research has revealed considerable nutritional and bioactive potential. However, the study of the bioactivity resulting from the combination of the two components to obtain an enriched flour is still poorly documented.

We therefore studied the overall glycemic profile of these flours, in particular their composition in resistant starch, amylose, and fiber, as well as their in vivo glycemic index. We also studied their bioactive content, in particular phenolic compounds and carotenoids, in relation to their antioxidant and anti-inflammatory potential.

Our results confirmed the predominance of carbohydrates in conventional cassava flour (CF), with a free sugar content below 2% and a proportion of starch equivalent to 82% of the flour. These findings are in accordance with the literature [61] reporting starch contents ranging from 74% to 81% in 25 different cassava varieties. Other work, such as that by Dudu et al., also confirmed a high starch content of 82.9% [62]. With regard to amylose content, our results, showing a 20.4% content, remain comparable to previous work that reported contents ranging from 13.1% to 24.8% [62,63,64]. When considering resistant starch, our study revealed a content of 13.14%, slightly lower than that reported by Aprianita et al. (19.3%), but significantly higher than previous measurements ranging from 5.7% to 7.07% [65]. Conversely, flour made from *Manihot esculenta* roots combined with 30% leaves revealed a decrease in carbohydrate content, combined with a significant increase in fiber content as expected. These observations corroborate our results about the physicochemical composition of leaves negligible carbohydrate content, i.e., <1%), as well as the literature data indicating high fiber contents in leaves, ranging from 8.41% to 16.36% [46]

In addition, previous studies have reported substantial amounts of protein in cassava leaves, ranging from 16.6% to 39.9% [11,46], covering the recommended daily protein intake (48 to 62 g) with a consumption of 500 g per day. Thus, these leaves are a superior source of protein when compared to conventional tropical legumes [12]. It is therefore relevant to note that the decrease in the proportion of starch in CFL flour can be explained by the increase in other nutrients provided by cassava leaves.

These changes in the physicochemical profile of flours have a direct impact on the glycemic index (GI). The latter is a criterion for classifying carbohydrate-containing foods. Numerous studies have demonstrated the importance of this parameter with regard to the development and prophylaxis of cardiovascular disease, diabetes, and obesity [66,67,68]. Indeed, it has been reported that high-GI diets stimulate de novo lipogenesis and lead to an increase in adipocyte size. Whereas, low-GI diets improve lipid profiles and increase insulin sensitivity [69].

GI is influenced by a variety of factors, including starch structure, physical configuration, plant matrix processing, as well as the nature and proportion of other macronutrients present in foods. In our study, we used the same cassava flour for both CF and CFL, so as to isolate differences in results solely linked to the addition of leaves. Our results suggest that CFL generated a reduced GI compared with CF, i.e., it induced a smaller rise of glycemia upon administration. In addition, the glucose load was reduced, with blood glucose levels reaching 153.4 mg/dL for CF and no more than 115.5 mg/dL for CFL. Studies on resistant starch and amylose confirm their involvement in the reduction in postprandial glycemia and insulin response [70,71]. Nonetheless, The measurements obtained in our study reveal comparable percentages of total starch across all the flours analyzed. In contrast, the fiber content is markedly higher in CFL, which is notable since increased dietary fiber consumption has been linked to improved glycemic control, a reduced glycemic index, and a decreased risk of cardiovascular disease. [72,73,74]. The addition of fiber from cassava leaves to flour induced a significant reduction in the latter’s glycemic index. This observation is consistent with results presented in the scientific literature. Indeed, previous studies by Saragih et al. [60] reported a similar trend when enriching wheat- and cassava-based cookies with leaves. By slowing the digestion of starch and delaying gastric emptying, fiber helps to reduce the glycemic response [74,75].

Increased fiber intake is closely associated with numerous health benefits. Evidence abounds in the literature in favor of a positive correlation between dietary fiber intake and insulin sensitivity [75,76,77,78,79,80]. In addition, research has shown an inverse relationship between total dietary fiber intake, including insoluble fiber, and the risk of cardiovascular disease [81]. In addition, studies have linked lower levels of C-reactive protein (CRP), a marker of systemic inflammation, to increased dietary fiber consumption [82]. The benefits of dietary fiber also include its ability to attenuate inflammation and regulate the immune response in the context of inflammatory bowel disease [83]. Thus, our results highlight a significant improvement in the nutritional potential of cassava flour thanks to fiber enrichment, accompanied by an improvement in the glycemic profile through reduction in the total proportion of carbohydrates and a lower glycemic index.

In addition to the macromolecules mentioned above, our study reveals a significant enrichment of cassava flour in carotenoids, more specifically beta-carotene and lutein. These compounds were absent in conventional flour (CF). These results are fully in line with literature reports, which also indicate an absence of carotenoids in non-biofortified cassava flour. However, it should be noted that increasing the amount of provitamin A carotenoids in cassava roots through selection and genetic modification is one of the current objectives in research for the nutritional improvement of this staple food crop in developing countries [84,85]. Vitamin A deficiency is widespread in sub-Saharan Africa, particularly in Nigeria, the world’s largest cassava producer, where 83% of children from age 2 to 5 are vitamin A-deficient [86,87,88,89]. Therefore, our results clearly demonstrate that enriching cassava flour with dried leaves leads to a significant increase in carotenoid content. These findings are in line with previously reported results about cassava leaves’ composition, which revealed the presence of lutein and beta-carotene at relatively high levels [14,46]. Indeed, our 30% addition of cassava leaves to flour generated a respective contents of 7.82 mg/100 g for lutein and 10.53 mg/100 g for beta-carotene. It would correspond to similar contents for the same amount of fresh spinach material in lutein [90,91], as well as to 50% of the beta-carotene content of an equivalent amount of orange sweet potato flour [58].

Beyond its conversion into vitamin A, beta-carotene is of particular interest due to its well-documented bioactive properties [87,92,93]. Indeed, studies have established a negative correlation between plasma beta-carotene levels and insulin resistance, as well as a positive correlation with plasma adiponectin levels, suggesting a beneficial impact of beta-carotene on insulin sensitivity [94]. As for lutein, various positive health effects were demonstrated, notably in pathological contexts such as age-related macular degeneration, or inflammatory and cardiovascular diseases [95,96].

Regarding conventional cassava flour mineral composition, our results are consistent with previous work [62,97]. The addition of cassava leaves contributed to improve CF minerals levels. Studies on leaf composition confirm our findings on enriched (CFL). For example, the study by Alamu et al. revealed significant quantities of iron, calcium, magnesium, and zinc in leaves [98]. Similarly, the study by Montagnac et al. indicated that calcium content in leaves is 100 times higher than in roots and that leaves are richer in iron, potassium, magnesium, copper, zinc, and manganese than roots [99]. These mineral contributions are particularly important in regions where cassava consumption is high. Indeed, Gegios et al.’s study on Nigerian children suggests that the proportion of cassava in the diet is inversely correlated with dietary intake of zinc, iron, and vitamin A [6]. Taken together, these data highlight the fact that regular inclusion of cassava leaves in the diet, at the same frequency as flour, could help reduce mineral deficiencies, particularly in essential elements such as iron, calcium, and zinc.

Phenolic compounds are molecules of known interest as bioactive compounds. They are associated with various effects on human health [100]. Numerous studies reported anti-inflammatory and antioxidant properties that could contribute to prophylactic and/or therapeutic effects in metabolic and cardiovascular diseases [43,101]. The antioxidant effects of polyphenols are based on various mechanisms that vary according to compounds’ molecular structure. These include the scavenging of free radicals such as OH^−^ and NO^−^ by polyhydroxyl and aromatic groups, or the chelation of transition metals in Fenton reactions, reducing radical production from H_2_O_2_. Our results indicate that cassava leaf powder provides a significant amount of phenolic compounds to conventional cassava flour (CF). Indeed, using the TPC test, we observed a fivefold higher content in the enriched flour (CFL) when compared to conventional flour (CF). These results are consistent with previous studies on the micronutrient composition of cassava leaves. For example, Laya et al. described the presence of a number of phenolic acids, such as *p*-hydroxybenzoic acid, *p*-catechuic acid, syringic acid, gallic acid, vanillic acid, benzoic acid, salicylic acid, and gentisic acid [45]. Other studies reported the presence of flavonoids, notably rutin in significant quantities [102].

According to our study, *Manihot esculenta* leaves contribute to improve CF content in compounds of nutrition–health interest. Compounds such as polyphenols, carotenoids, fibers, and minerals, in addition to tuber macromolecules’ profile (e.g., resistant starch) exert beneficial and sought-after bioactivities for human health, notably anti-inflammatory and antioxidant activity, as studied in this work [41,44,103,104].

Modulation of radical oxygen species (ROS) production in the human body is recognized as a major therapeutic objective for the management of various pathologies, including non-communicable diseases such as metabolic syndrome, obesity, type 2 diabetes, hypertension, as well as for certain forms of cancer and neurodegenerative diseases [37,43,105]. In our study, results obtained with conventional cassava flour (CF) revealed a relatively low antioxidant capacity. However, the addition of cassava leaves significantly enhanced this activity. Indeed, our data showed a concentration-dependent nitric oxide (NO) scavenging capacity, on average, 4.5 times higher in CFL when compared to CF. NO is a free radical involved in oxidative stress and acts as a pro-inflammatory mediator in acute and chronic inflammatory processes. The mechanism by which this radical is trapped is still poorly understood. However, a study was carried out on 31 medicinal plants to assess their possible regulatory effect on NO levels using sodium nitroprusside as an in vitro NO donor. Eight of them demonstrated an ability to neutralize NO directly, and of these, seven were found to contain tannins as their main constituents. In the same study, the ten main tannins contained in these plants showed high NO scavenging activity. Six of the eight alkaloids obtained from one of the samples also effectively scavenged this radical, suggesting that these compounds could be active principles responsible for NO scavenging in foods [106]. Additional reports described an important NO scavenging capacity of other phenolic compounds, notably flavonoids and tannins [107]. The presence of flavonoids, tannins, and saponins in cassava tubers was described [108]. The presence of tannins, saponins, terpenoids, flavonoids, carotenoids, and phenolic acids has also been demonstrated in cassava leaves [109,110]. These data are also in line with the work of Fioroni et al., describing a 76% inhibition of NO by a cassava leaf extract [111].

The DPPH test simulates the production of free radicals in order to assess the ability of a compound or extract under investigation to supply a hydrogen atom. Our results reveal a significant radical scavenging capacity of CFL extract, while a much lower scavenging capacity was recorded for CF extract. Therefore, the enriched flour acquires antioxidant properties via this leaf powder addition without observable antagonist influence.

These results for CFL extract are consistent with literature findings describing a DPPH scavenging capacity of 84 µmol TE per gram of a polar extract of cassava leaf [111]. As far as cassava flour is concerned, few studies have evaluated its biological activities, including antioxidant capacity. Although some previous research produced results, their direct comparison with the results of the present study is limited due to the nature of the extract (dry, liquid, or fraction) [97,112]. However, our results clearly indicate a strong increase in antioxidant properties associated to the addition of cassava leaves powder. Indeed, our results for the DPPH test revealed an antioxidant activity on average 5.5 times higher in (CFL) than in (CF). Thus, the CFL extract showed a significantly increased capacity to neutralize NO and DPPH radicals, inhibiting oxidative aggression by 58.76% and 60.47%, respectively, while the conventional flour extract (CF) achieved only 11.22% and 11.96% for these two measures.

Results were further validated by the ORAC assay, a standard method for determining food antioxidant capacity by assessing the neutralization of free radicals, such as peroxides. In our analysis, the antioxidant capacity measured by ORAC for CFL was 1626.17 µmol TE/g of dry weight, significantly higher than the 457.30 µmol TE/g dry weight recorded for CF. Clearly, plant green parts/leaves demonstrate greater antioxidant properties than the starchy parts. These findings are in line with those reported in the literature. By way of comparison, spinach, cassava leaves, and refined wheat flour were reported with ORAC values of 734, 3083.16 and 12.52 µmol TE/g [14,111,113,114].

The antioxidant activity observed in leaf-enriched flour can be attributed, among other factors, to the bioactive phytochemical compounds present in these leaves (carotenoids and phenolic compounds). These compounds are plant secondary metabolites that exert both direct and indirect antioxidant effects by stimulating cells’ endogenous antioxidant defense systems [115,116,117,118,119]. They are also recognized for their anti-inflammatory properties.

Inflammation and the resulting production of inflammatory cytokines are typical, non-specific immune responses triggered by biotic or abiotic challenges to the body. However, chronic inflammation is also a key factor in numerous human diseases, including non-communicable conditions such as metabolic syndrome, obesity, type 2 diabetes, cardiovascular diseases, cancer development, and neurodegenerative disorders. Phenolic compounds sourced from plant biodiversity have been identified as potent anti-inflammatory agents, effective both in vitro and in vivo [120,121,122,123]. Their mechanisms of action often involve the modulation of key signaling pathways and molecular targets, such as NF-kB, MAPk, PI3K/Akt, PLA2, and cyclooxygenases (COX), which ultimately reduce prostaglandin synthesis. Moreover, polyphenols can influence endogenous anti-inflammatory enzymes such as catalase, superoxide dismutase (SOD), and glutathione peroxidase [121,123]. Carotenoids also exhibit anti-inflammatory effects by modulating the NF-kB and MAPk pathways, potentially acting synergistically with polyphenols, as evidenced by clinical studies [124].

To explore and compare the anti-inflammatory and immunomodulatory potential of both our CF and CFL, we used endotoxin LPS-stimulated macrophages. LPS induces an increase in synthesis and production of various major inflammation mediators and cytokines, namely: nitric oxide (NO) radical, prostaglandin-E2 (PGE-2), Interleukin-6 (IL-6), Tumor necrosis factor-alpha (TNF-α) involved in the acute phases of inflammation and Monocyte Chemoattractant Protein-1 (MCP-1) involved in the cellular aspect of inflammation. We therefore exposed stimulated macrophages to CF and CFL in order to assess the inflammation markers’ production profile alterations.

CF and CFL did not alter macrophage cells’ viability and growth. This result demonstrates CF and CFL innocuity and cell biological safety. Inhibition of cell inflammatory marker production was improved several folds in CFL when compared to CF. Indeed, conventional cassava flour (CF) extract showed relatively moderate inhibition of NO radical and PGE-2 cell production, as well as negligible inhibition of the other cytokines’ secretion. In the case of flour enriched with 30% cassava leaves (CFL), our results showed an overall strong concentration-dependent inhibition of all inflammation biomarkers studied.

The findings regarding CFL align with the existing literature that highlights the immunomodulatory properties of *Manihot esculenta* leaf extracts in vivo. Several studies demonstrated the anti-inflammatory activities of these extracts. For instance, research by Adeyami et al. showed that cassava leaf extract can inhibit chemically induced acute inflammation in rodents when administered orally or topically [47]. Furthermore, Miladiyah et al. identified the analgesic effects of *Manihot esculenta* leaf extracts in mice, with an efficacy comparable to paracetamol [48].

Considering that pain reduction occurs alongside alleviation of inflammation in vivo, combined with the in vitro data, it suggests that the PLA2/COX/PG (prostaglandin) pathway might play a role, given that prostaglandins serve as mediators for both inflammation and pain [125].

As far as cassava flour, whether conventional (CF) or leaf-enriched (CFL) is concerned, to the best of our knowledge, it is the first time that (1) anti-inflammatory and immunomodulatory properties of this type of extracts are described and (2) a bioguided approach is used to optimize nutrition–health properties of a vegetal food matrix through recombination of its subparts.

## 5. Conclusions

The results of our study highlight the remarkable nutritional properties of cassava leaves and the significant benefits of enriching conventional flour (CL) with these leaves. A comparison of nutritional compositions between conventional (CF) and enriched cassava flour (CFL) reveals a noteworthy increase in fiber, minerals, phenolic compounds, and carotenoids, as well as an expected significant reduction in carbohydrate content in the enriched flour, resulting in a lower glycemic index.

Our data also highlight the improved bioactive properties induced by this bioguided enrichment, with significantly higher antioxidant and free radical scavenging power of the flour enriched with cassava leaves. The ability to neutralize nitric oxide—in a concentration-dependent manner—was four times greater in CFL than in CF.

Furthermore, our study also showed that the addition of 30% cassava leaves to flour induced a marked improvement in its ability to reduce inflammation, with several folds’ significant concentration-dependent inhibition of pro-inflammatory cytokines such as IL-6, TNF-alpha, and MCP-1. These effects were almost absent in conventional flour (CF). In addition, there was a moderate, still significant, improvement in the inhibition of nitric oxide (NO) and prostaglandin E2 (PGE-2).

Our results suggest that bioguided incorporation of 30% cassava leaves into cassava flour not only provides antioxidant, anti-inflammatory, and immunomodulatory properties, but also a non-carbohydrate nutritional contribution to reduce peak glycemia. This approach may support the prophylaxis and management of non-communicable metabolic diseases as well as nutritional imbalances.

In this perspective, further research is needed to (1) explore and compare the comparative effects of CF and CFL on preclinical models of metabolic syndrome with low-grade chronic inflammation status, (2) identify the qualitative and quantitative combinations of compounds responsible for these health benefits, (3) optimize the nutritional, sensory and bioactive qualities of the final products derived from the enriched flour (CFL), usually derived from conventional flours, and (4) formalize bioguided processes for optimization and prediction of complex vegetal-derived food matrixes’ nutrition–health effects. Our research group is actively engaged in these investigations, and preliminary results are promising.

## Figures and Tables

**Table 1:** Physicochemical composition of conventional cassava flour (CF) and enriched cassava flour (CFL). Glycemic index was assessed on *n* = 8 animals per group (CF and CFL). Values are expressed as mean plus or minus the standard deviation (SD) (*n* = 3) (^a^ and ^b^; *p* ≤ 0.05).

Composition and Glycemic Index of CF and CFL Samples			
		Samples	
		CF	CFL
Yield (%)		32.19 ± 2.24	-
Moisture (% flour)		8.32 ± 0.23 ^a^	7.01 ± 0.41 ^b^
Carbohydrates (% DM flour)		84.12 ± 2.36 ^a^	59.24 ± 1.88 ^b^
	Total starch	82.93 ± 2.66 ^a^	57.85 ± 1.98 ^b^
	Simple sugars	1.19 ± 0.03 ^a^	1.39 ± 0.04 ^a^
Amylose (% DM starch)		20.36 ± 1.4 ^a^	20.28 ± 1.00 ^a^
Resistant starch (% DM flour)		13.14 ± 3.51 ^a^	9.09 ± 0.87 ^b^
Total insoluble fibers (% DM flour)		3.65 ± 0.17 ^b^	15.20 ± 0.91 ^a^
	Hemicellulose	1.92 ± 0.25 ^b^	5.01 ± 0.61 ^a^
	Cellulose	1.60 ± 0.02 ^b^	3.40 ± 0.05 ^a^
	Lignin	0.13 ± 0.017 ^b^	6.79 ± 0.81 ^a^
Glycemic index		91.09 ± 1.75 ^a^	72.12 ± 3.25 ^b^

**Table 2:** Composition of CF and CFL in mineral matter, carotenoids, and total amount of phenolic compounds. Values are expressed as mean plus or minus SD (*n* = 3) (^a^ and ^b^: *p* < 0.05).

Composition of Potentially Bioactive Micronutrients				
Sample	TPC in mg GAE/g EDW	Minerals Matters (% DM/g‧100 g^−1^)	Carotenoids (% DM/mg‧100 g^−1^)	
			Lutein	β-Carotene
CF	15.16 ± 0.87 ^b^	2.4 ± 0.6 ^b^	-	-
CFL	86. 59 ± 3.09 ^a^	4.01 ± 0.48 ^a^	7.82 ± 1.02	10.53 ± 0.98

**Table 3:** Determination of antioxidant potential of flours (CF) and (CFL), by NO scavenging, DPPH, and ORAC tests. Values are expressed as mean plus or minus SD. Means with different superscripts within a column are significantly different (^a^ and ^b^: *p* < 0.05).

Antioxidant Activity					
Samples	NO Scavenging		DPPH		ORAC
	Inhibition (%) at 100 µg/mL	Inhibition (%) at 50 µg/mL	µmol TE/g EDW	Inhibition (%) at 1 mg/mL	µmol/TE/g EDW
CF	11.22 ± 1.93 ^b^	7.83 ± 1.13 ^b^	15.62 ± 0.93 ^b^	11.96 ± 0.65 ^b^	457.30 ± 7.72 ^b^
CFL	58.76 ± 2.01 ^a^	30.66 ± 1.63 ^a^	93.49 ± 7.32 ^a^	60.47 ± 1.17 ^a^	1626.17 ± 202.45 ^a^

## Data Availability

Data are contained within the article.
